# Targeted-Alpha-Therapy Combining Astatine-211 and anti-CD138 Antibody in a Preclinical Syngeneic Mouse Model of Multiple Myeloma Minimal Residual Disease

**DOI:** 10.3390/cancers12092721

**Published:** 2020-09-22

**Authors:** Sébastien Gouard, Catherine Maurel, Séverine Marionneau-Lambot, Delphine Dansette, Clément Bailly, François Guérard, Nicolas Chouin, Ferid Haddad, Cyril Alliot, Joëlle Gaschet, Romain Eychenne, Françoise Kraeber-Bodéré, Michel Chérel

**Affiliations:** 1 Université de Nantes, CNRS, Inserm, CRCINA, F-44000 Nantes, France; sebastien.gouard@univ-nantes.fr (S.G.); catherine.sai-maurel@inserm.fr (C.M.); francois.guerard@univ-nantes.fr (F.G.); nicolas.chouin@oniris-nantes.fr (N.C.); alliot@arronax-nantes.fr (C.A.); joelle.gaschet@univ-nantes.fr (J.G.); Romain.eychenne@univ-nantes.fr (R.E.); 2 Department of nuclear medicine, Université de Nantes, CHU de Nantes, CNRS, Inserm, CRCINA, F-44000 Nantes, France; severine.marionneau-lambot@univ-nantes.fr (S.M.-L.); clement.bailly@chu-nantes.fr (C.B.); francoise.bodere@chu-nantes.fr (F.K.-B.); 3 Centre Hospitalier Départemental de Vendée, F-85925 La Roche-Sur-Yon, France; delphine.dansette@chd-vendee.fr; 4 ONIRIS, F-44000 Nantes, France; 5 Université de Nantes, IMT Atlantique, CNRS, Subatech, F-44000 Nantes, France; haddad@subatech.in2p3.fr; 6 Groupement d’Intérêt Public Arronax, F-44800 Saint-Herblain, France; 7 ICO-René Gauducheau Cancer Center, F-44800 Saint-Herblain, France

**Keywords:** Targeted alpha therapy, astatine-211, multiple myeloma, CD138, minimal residual disease

## Abstract

**Simple Summary:**

Multiple myeloma is a cancer that remains incurable. Among the many therapies under evaluation, antibodies can be used as vehicles to target and deliver toxic radiation to the tumour cells. Our objective was therefore to investigate the potential of targeted alpha therapy, combining an anti-CD138 mAb with astatine-211, to destroy the residual cells responsible for relapse. We have shown in a mouse model that mimics human disease, that destroying multiple myeloma cells is feasible with low toxicity by injecting an anti-CD138 mAb coupled with astatine-211. This approach could eradicate residual cells after initial treatment and thus prevent recurrence.

**Abstract:**

Despite therapeutic progress in recent years with the introduction of targeted therapies (daratumumab, elotuzumab), multiple myeloma remains an incurable cancer. The question is therefore to investigate the potential of targeted alpha therapy, combining an anti-CD138 antibody with astatine-211, to destroy the residual cells that cause relapses. A preclinical syngeneic mouse model, consisting of IV injection of 1 million of 5T33 cells in a KaLwRij C57/BL6 mouse, was treated 10 days later with an anti-mCD138 antibody, called 9E7.4, radiolabeled with astatine-211. Four activities of the ^211^At-9E7.4 radioimmunoconjugate were tested in two independent experiments: 370 kBq (*n* = 16), 555 kBq (*n* = 10), 740 kBq (*n* = 17) and 1100 kBq (*n* = 6). An isotype control was also tested at 555 kBq (*n* = 10). Biodistribution, survival rate, hematological parameters, enzymatic hepatic toxicity, histological examination and organ dosimetry were considered. The survival median of untreated mice was 45 days after engraftment. While the activity of 1100 kBq was highly toxic, the activity of 740 kBq offered the best efficacy with 65% of overall survival 150 days after the treatment with no evident sign of toxicity. This work demonstrates the pertinence of treating minimal residual disease of multiple myeloma with an anti-CD138 antibody coupled to astatine-211.

## 1. Introduction

Symptomatic multiple myeloma (MM) is a malignant gammopathy characterized by an abnormal abundance of monoclonal plasma cells within the bone marrow. With ~150,000 new cases per year diagnosed in the world (source: World Health Organization), MM represents 1.9% of all cancers [1] and is the second most common blood cancer.

Thanks to new therapeutic regimens based on combinations of several classes of drugs (including proteasome inhibitors and immunomodulatory drugs) and the benefit of autologous stem cell transplant for eligible patients, median survival has been increased by 2 years in the past decade [2,3]. However, MM remains incurable because current treatments are unable to eradicate all malignant cells, notably because of drug resistance [4]. To overcome these resistances, immunotherapeutic strategies based on targeted therapies with monoclonal antibodies have been approved in 2015 for targeting SLAMF7 with elotuzumab or CD38 with daratumumab [5]. Although these therapies are gaining in importance, they are still mainly administered on relapsed or refractory multiple myeloma patients. While these therapies bring patients benefits, some resistances have also been reported [6].

In this context where relapse is inevitable, our strategy is to eradicate the minimal residual disease (MRD) using a monoclonal antibody as a vector of an α-particle-emitting radionuclide. This strategy is called Targeted-Alpha-Therapy (TAT). The chosen targeted antigen is CD138, also called syndecan-1, which is a proteoglycan expressed on epithelial cells and broadly overexpressed on the extracellular membrane of myeloma cells [7,8,9]. CD138 plays a major role of cell survival by participating in cell adhesion and cell proliferation [10]. 

Alpha particles seem to be particularly suitable to achieve this objective. Indeed, their path length of around 70 µm in tissue and their high linear energy transfer (~100 keV/µm) confer to α-particles a high cytotoxic potential suited to eradicating isolated cells or small clusters of cells [11,12,13]. Among α-particle-emitting radionuclides, only a few are relevant for clinical application considering their half-lives and their ability to be stably conjugated to a biomolecule. Furthermore, the supply of certain radionuclides could be a problem, notably for actinium-225 and, consequently, for bismuth-213 whose global production is currently very limited. Astatine-211 provides the advantage of being produced in cyclotron by irradiation of a natural bismuth target and, according to Lindegren et al. [14], around 30 cyclotrons in the world are fit for its production. Moreover, astatine-211 exhibits a half-life of 7.2 h which is appropriate with antibody biodistribution and is theoretically more suitable than bismuth-213 and its significantly shorter half-life of 45 min. To our knowledge and to date, five clinical trials (NCT04083183, NCT03128034, NCT03670966, NCT00003461) [15,16,17] with astatine-211 have been approved, following promising preclinical studies conducted on mice [18,19,20]. The aim of this new study was to evaluate the efficacy of an anti-mCD138 antibody radiolabeled with astatine-211 to treat MRD in a syngeneic preclinical model of MM. This study is a follow-up of our work carried out with bismuth-213 [21,22].

## 2. Results

### 2.1. Anti-CD138 Antibody, ^211^At Radiolabeling, Immunoreactivity and Flow Cytometry

As previously described by Fichou et al. [22], the 9E7.4 anti-mCD138 mAb was the result of a rat immunization with a 40-amino-acid peptide issued from the extramembranous part of murine syndecan-1. Its specificity has been studied by ELSA assay, cytometry [22] and by immunohistochemistry on skin tissue, spleen tissue, liver tissue and lung tissue. This antibody has also been used with PET imaging to detect MM subcutaneous and intramedular lesions [23,24].

**Figure 1:**
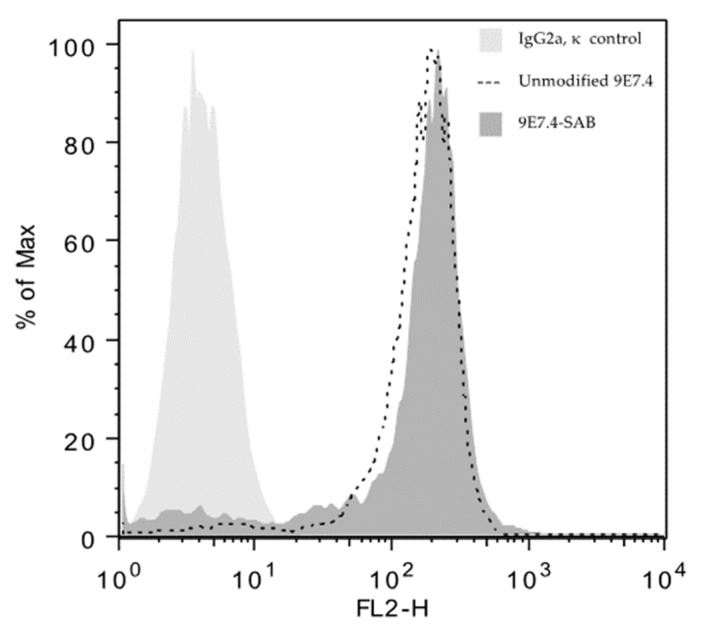
Flow cytometry data on 5T33 MM (multiple myeloma) cells showing the specific binding of unmodified 9E7.4 and modified 9E7.4 SAB (N-succinimidyl-3-[211At]astatobenzoate prosthetic group). Antibodies were incubated for 1 h and revealed with an anti-rat secondary antibody labeled with phycoerythrin.

Three independent productions of the N-succinimidyl-3-[211At]astatobenzoate prosthetic group (SAB) were performed using aryliodonium salt chemistry [25], and conjugations on the 9E7.4 anti-mCD138 antibody and an isotype control mAb were achieved with respective conjugation yields of 68 ± 2% (*n* = 3) and 65% (*n* = 1). After purification using a size exclusion chromatography column, the radiochemical purity of ^211^At-9E7.4 mAb and ^211^At-isotypic control mAb was greater than 99%. The immunoreactivity of ^211^At-9E7.4 mAb was 83.0 ± 3.0% and in parallel, after radioactive decay, the specific binding of ^211^At-9E7.4 mAb was validated by cytometry on 5T33 MM cells (Figure 1). The results show that the radiolabeling process did not affect the ability of the mAb to bind the targeted cells.

### 2.2. Biodistribution Study

**Figure 2:**
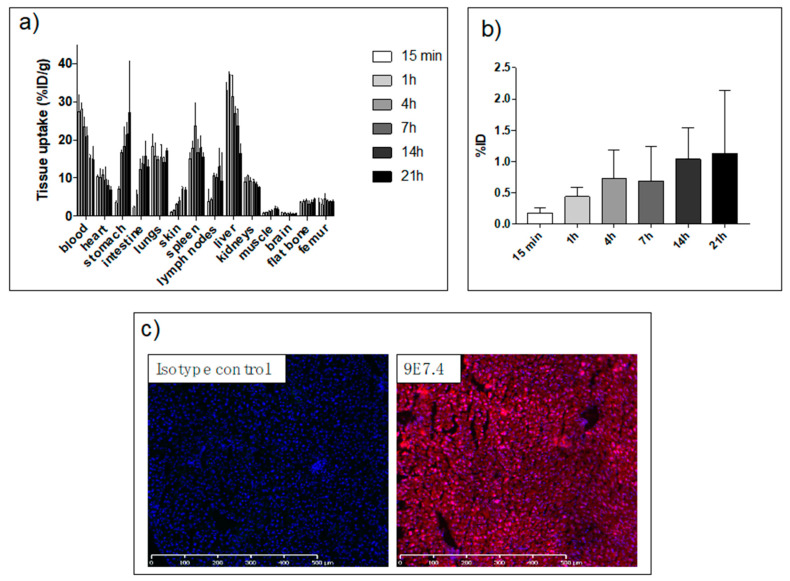
Biodistribution data analysis of the immunoconjugate ^211^At-9E7.4 and immunofluorescence data of CD138 expression on liver tissue. (**a**) Biodistribution data of ^211^At-9E7.4 mAb. Three animals were sacrificed at each time point. Data are expressed at the percentages of injected dose per gram of tissue (%ID/g). Means and SD are depicted. (**b**) Biodistribution data of ^211^At-9E7.4 mAb in thyroid. Three animals were sacrificed at each time point. As it is difficult to collect the thyroid, the neck was collected, counted and the data are expressed at the percentages of injected dose (%ID). Means and SD are depicted. (**c**) Immunofluorescence analysis of liver section with 9E7.4 or isotype control IgG2a, κ labeling with Alexa Fluor 647(pink). Nuclei are visualized with DAPI (blue). Scans were performed using a slide-scanner Nanozoomer (Hamamatsu^®^).

We previously reported biodistribution data of 9E7.4 [22,23,24] but it was radiolabeled with other radionuclides (lutetium-177, copper-64 and zirconium-89). Considering the potential impact of the radionuclide and its radiolabeling method, a biodistribution study with ^211^At-9E7.4 mAb has been performed (Figure 2a). Sacrifices were conducted at 15 min, 1 h, 4 h, 7 h, 14 h and 21 h post-administration of ^211^At-9E7.4 mAb on C57BL/KaLwRij mice (3 mice/group). Several occurrences were observed. The first one was linked to the nature of the vector and its persistence in the blood compartment (14.9 ± 4.5% ID/g at 21 h) and in highly perfused tissues. The second one was linked to the deastatination of radiolabeled mAb. Because of similarities in behavior between astatine and iodine, two halogens, an iodine solution (called Lugol’s solution) was administrated before injection of radioimmunoconjugate [26]. So, the uptake of free ^211^At was low in the thyroid (Figure 2b). However, radioactivity increased over time in the lung, stomach and intestine due to the dehalogenation and the inability of Lugol’s solution to block free astatine in these organs [26]. The third phenomenon is associated with the specific binding of 9E7.4 mAb to CD138 and specific pharmacodynamic behavior was observed for spleen and skin. For the liver, a particular distribution was observed with a high level of 33.0 ± 2.2%ID/g at 15 min followed by a progressive decrease from 4h to 21h to 16.4 ± 2.5%. Liver uptake was investigated by immunofluorescence assay in order to evaluate the uptake’s part due to the Fc binding. Figure 2c shows that, unlike 9E7.4, isotype control mAb was unable to bind liver’s frozen sections. Therefore, the liver’s uptake appeared to be the result of a specific binding.

### 2.3. ^211^At-anti-mCD138 TAT in a Disseminated Murine MM

**Figure 3:**
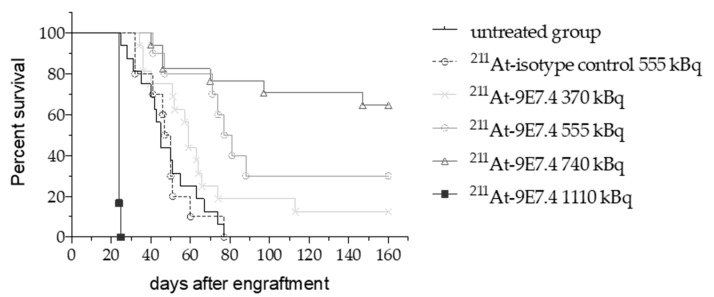
Kaplan–Meier survival curves of TAT (Targeted-Alpha-Therapy) in a disseminated MM mouse model. C57BL/KaLwRij mice received 10^6^ 5T33 MM cells followed 10 days later by injection of ^211^At-9E7.4 at 370 (*n* = 16), 555 (*n* = 10), 740 (*n* = 17) and 1110 (*n* = 6) kBq. Control mice received ^211^At-IgG2a isotype control at 555 kBq (*n* = 10) or no treatment (*n* = 16). Data were issued from 2 independent experiments.

We have assessed the efficacy of ^211^At-9E7.4 in a mouse model of MRD in MM. The experimental model was obtained 10 days after intravenous injection of 10^6^ 5T33 MM cells in C57BL/KaLwRij mice. At this time point, secreted immunoglobulins were undetectable and intramedullary lesions were not yet detectable with PET imaging [21,24]. Then 370, 555, 740 or 1110 kBq of ^211^At-9E7.4 mAb was injected intravenously into 16, 10, 17 and 6 mice, respectively. A group of 10 mice was injected with isotype control IgG2a, κ with an activity of 555 kBq (Figure 3). Sixteen mice received no treatment and showed a median survival of 45 days. Mice were euthanized when they became moribund, when a weight loss of more than 20% was measured, when signs of paraplegia were observed or when extramedullary lesions were visible. Death causes are listed in Table 1. Studies demonstrated a highly statistically significant survival benefit for the mice treated with ^211^At-9E7.4 at 555 kBq (*p* = 0.0006) and 740 kBq (*p* < 0.0001). At 555 kBq, the median survival was increased by 34 days and at 740 kBq 11/17 mice survived for 160 days after engraftment. For treatments with ^211^At-9E7.4 at 370 kBq or ^211^At-isotype control at 555 kBq no significant benefit was observed. The highest activity with 1110 kBq of ^211^At-9E7.4 was clearly radiotoxic. All mice were euthanized after a drastic weight loss superior to 20% or initial weight 14 days after radiopharmaceutical injection. Except for this weight loss, no other macroscopic signs of toxicity have been revealed during the autopsy. 

### 2.4. Monitoring of Early Toxicity of the Treatment

**Figure 4:**
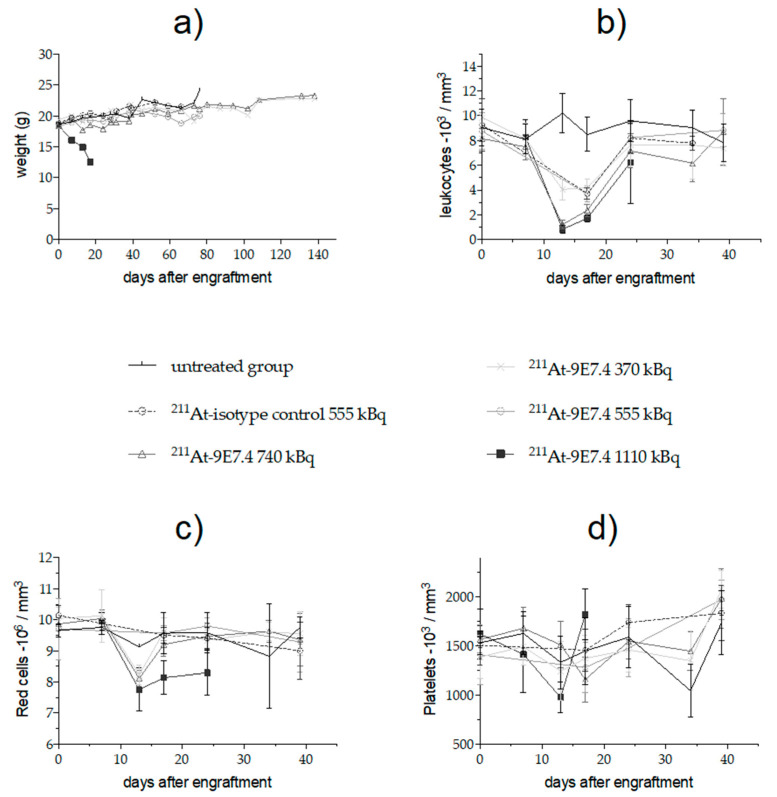
Weight (**a**) and hematologic toxicity of mice grafted with 10^6^ 5T33 MM cells then treated with ^211^At-mAb. Leucocytes (**b**), red blood cells (**c**) and platelets (**d**) were analyzed using quantitative automated hematology analyzer and are given as means and SD of surviving mice.

Besides the deterioration of the general condition, the group receiving 1110 kBq of ^211^At-9E7.4 exhibited a severe but transient leukopenia 3 days after treatment injection and a high but transient decrease (39.0% ± 7.8%) of platelets 10 days after treatment injection (Figure 4). Red blood cells were impacted too and, in contrast to leukocytes and platelets, their levels were low until the mice died. For the other groups, transient decreases of leukocytes proportionally to the injected activities were measured (69.9% ± 6.3%, 56.1% ± 14.7%, 53.6% ± 7.5% at 740, 555 and 370 kBq, respectively). A leucocyte drop was also observed in the isotype control due to the persistence of the antibody into the bloodstream. As for leukocytes, the level of red blood cell decrease was transient and proportional to the injected activity.

### 2.5. Monitoring of Late Toxicity of the Treatment by ASAT (Aspartate Aminotransferase) and ALAT (Alanine Aminotransferase) Enzymatic Assay

**Figure 5:**
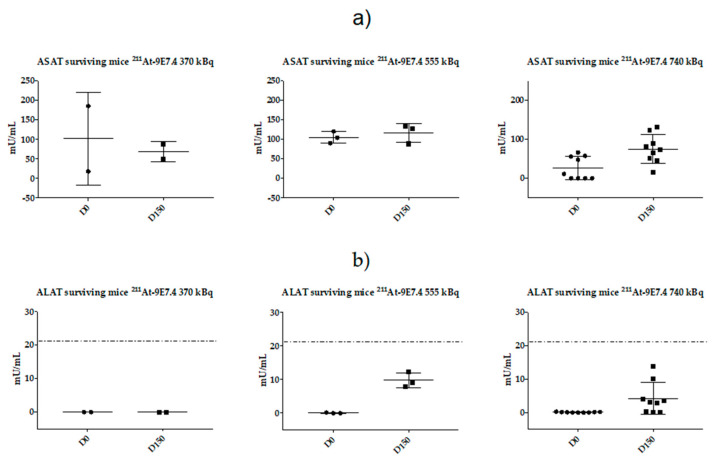
Hepatic toxicity of mice grafted with 10^6^ 5T33 MM cells then treated with ^211^At-9E7.4. ASAT (aspartate aminotransferase) (**a**) and ALAT (alanine aminotransferase) (**b**) were measured on serum from all surviving mice 150 days after engraftment. Dashed lines on ALAT graphs indicated the upper normal limit for ALAT parameter (issued from [27]).

In surviving mice, no clinical signs were visible during the follow-up period (weight, Figure 4a). However, since the radiopharmaceutical presented a high distribution into the liver within the first hours, we evaluated the long-term toxicity by ASAT (aspartate aminotransferase) and ALAT (alanine aminotransferase) measurement on surviving animals at the end of the study (Figure 5). Our previous study [21] had demonstrated the relevance of studying these parameters in TAT. There is no significant difference for ASAT level at D150 between the three injected activities (370 kBq, 555 kBq and 740 kBq) while ALAT levels were increased in the 555 kBq and 740 kBq groups but remained into the normal ranges described in the literature for C57Bl6 mice [27].

### 2.6. Monitoring of Late Toxicity of the Treatment by Histology

#### 2.6.1. Histological Examination of the Liver

**Figure 6:**
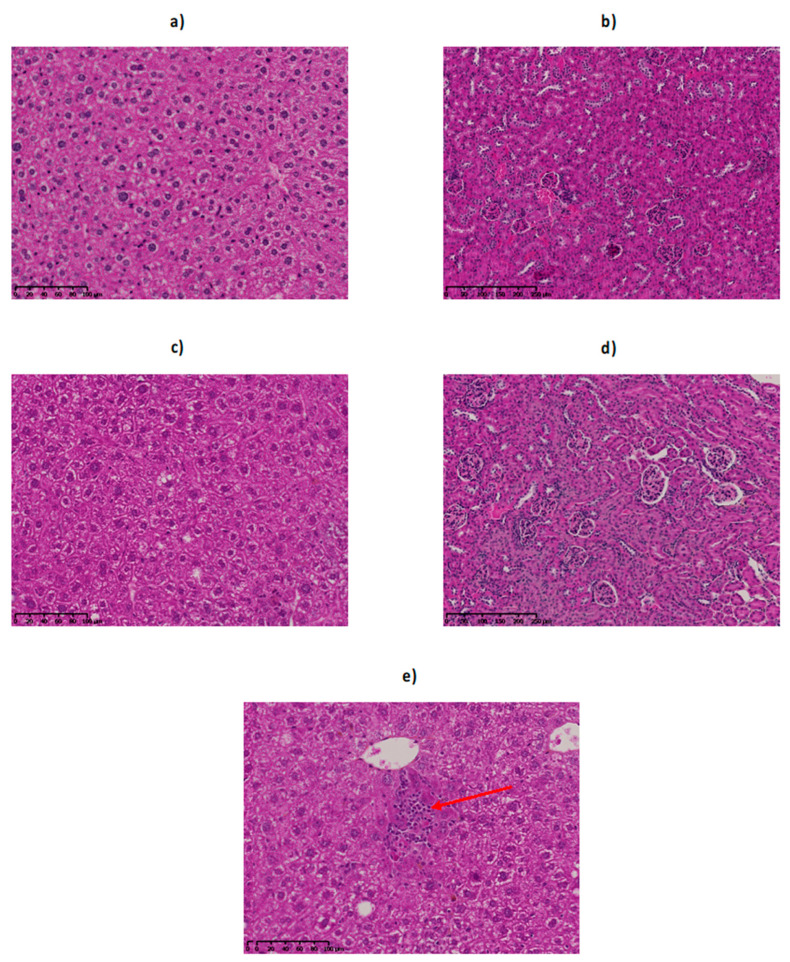
Liver and kidney histology of control and ^211^At-9E7.4 injected mice. Control liver (**a**) and kidneys (**b**), as well as livers (**c**) and kidneys (**d**) of mice 160 days after injection of 740 kBq of ^211^At-9E7.4 were histologically examined. No significant histological injury was observed. Livers of mice injected with ^211^At-9E7.4 show extramedullary hematopoiesis (**e**, arrow), yet in a similar proportion to control mice. Hematoxylin-Phloxin-Saffron.

Macroscopic inspection of animal’s livers after sacrifice as well as histologic examinations did not show any sign of toxic effects of all injected activities (Figure 6a,c,e). Classic radiation-induced abnormalities [28], such as cytomegaly, karyomegaly, intra-nuclear cytoplasmic invagination, inflammation and fibrosis were not observed. Foci of extramedullary hematopoiesis (EMH) were encountered in all injected mice. EMH is characterized by groups of dozens of hematopoietic precursors randomly distributed in the hepatic sinusoids as well as around central veins and in portal areas. EMH could be the reflection of radiation-induced myelotoxicity and failure of central hematopoiesis, yet it is also commonly found in rodents’ liver in physiological conditions. In our study, EMH was found in both control and injected mice in similar proportions. 

#### 2.6.2. Histological Examination of the Kidney

Kidneys of injected rodents showed no histological alterations (Figure 6b,d). Classic radiation-induced abnormalities [29], such as cellular atypies, proteinaceous casts, glomerulosclerosis or tubular injuries were not observed.

#### 2.6.3. Histological Examination of the Spleen

**Figure 7:**
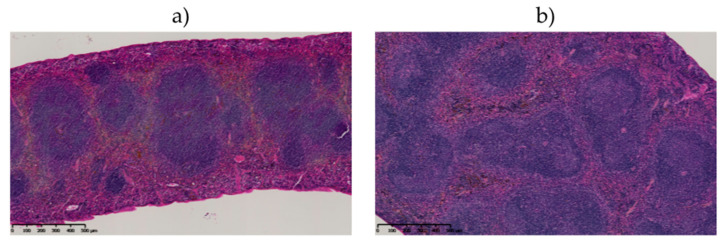
Spleen histology of control and ^211^At-9E7.4 injected mice. Comparison between spleen of control rodent (**a**) and of mice 160 days after injection of 740 kBq of ^211^At-9E7.4 (**b**) showing lymphoid follicular hyperplasia in this latter. Hematoxylin-Phloxin-Saffron.

Only organs of mice injected with 740 kBq of ^211^At-9E7.4 showed histological changes as lymphoid follicular hyperplasia was observed (Figure 7). This reactive response yet appears non-specific and classic radiation-induced alterations, such as lymphoid depletion in the white pulp or diffuse fibrosis of the red pulp, were not observed in any group.

### 2.7. Dosimetry Study

Based on the ^211^At-9E7.4 biodistribution depicted in Figure 2, the blood absorbed dose was calculated to be 8.0 Gy/MBq (+/− 0.7). For liver, spleen and kidney, absorbed doses were found to be 10.9 (+/− 1), 8.2 (+/− 1.7) and 3.6 (+/− 0.3) Gy/MBq. With the most efficient activity of 740 kBq, the absorbed doses are 6.0, 8.0, 6.1, 2.7 Gy for blood, liver, spleen and kidneys. All dosimetry results are listed in Table 2.

## 3. Discussion

Using a syngeneic mouse model of MM, we demonstrated the efficacy of combining an anti-CD138 antibody with astatine-211, a radioisotope that emits alpha particles. In this disseminated 5T33 MM model, where the median survival was 45 days without treatment, the 740 kBq activity appeared to be the most efficient with nearly 65% of mice having no clinical signs of pathology 150 days after the graft and no treatment-induced toxicity. Because of the aggressivity of the 5T33-induced MM model, where only 500 cells can lead to a paraplegia 40 days after engraftment [30], we can consider that mice were cured by TAT. This study confirms the potential of TAT demonstrated previously with another alpha particle emitter, bismuth-213 [21], which outperforms the clinical treatments based on chemotherapy (Melphalan, Bortezomib) studied in this model [31,32].

The study was conducted in the specific context of MM where, despite the addition of new molecules to the therapeutic arsenal, the pathology is still considered as incurable [33]. Drug resistance through adaptive mechanisms is the major reason for this, with an almost inevitable relapse that will have to be treated with new approaches [34,35,36]. To avoid these relapses, the idea would be to eliminate the residual cancer cells by using TAT after the first lines of treatment. Indeed, few resistances to alpha particles have been reported in the literature due to their high cytotoxic potential [37,38,39]. In vitro experiments conducted on a 5T33 cell line demonstrated that alpha particles induce DNA double strand breaks followed by cell proliferation arrest and a cell cycle blockade in G2 phase, finally leading to necrosis in most cases [40]. Furthermore, we observed in more than 10 human MM lines that whatever their cytogenetic status (p53 status, translocations...), they were all sensitive to alpha particles. Indeed, with a linear energy transfer around 100 keV/µm, it has been shown that a small number of alpha particles crossing an isolated cell were sufficient to cause cell death [41]. Because of this high cytotoxic potential, radionuclides must be stably driven as close as possible to the targeted cells. The SAB prosthetic group was chosen for its simplicity and the low impact of this radiolabeling approach on antibody pharmacokinetic profiles. However, with regard to the uptake observed in the lungs and stomach due to the dehalogenation of astatine-211 (Figure 2a) it is clear that there is room for improvement of the radiolabeling strategy with this radionuclide.

For the targeting of malignant cells, a therapeutic approach with antibodies seems to be appropriate, with the 7.2-h half-life of astatine-211 and the antibodies’ robustness with high affinity which have been proven. For the antigenic target, several surface molecules have been explored as potential targets of mAb, including SLAMF7 (CS1), CD38, CD40, CD138, CD56, CD54, IL-6, PD1, CD74, CD162, β2-macroglobulin, and GM-2 [42]. We have particularly considered CD38 or SLAMF7 which are the subject of immunotherapies that have received approval in 2015 for the treatment of MM [43]. However, the expression of these molecules on the surface of many immune cells [44,45,46] would a priori lead to a significant irradiation of secondary lymphoid organs. However, this approach has been investigated by Quelven et al. and O’Steen et al. [47,48] using an anti-CD38 antibody coupled respectively with ^212^Pb and ^211^At as alpha particle emitters. However, preclinical therapies were conducted on a xenograft model which is not suitable for assessing toxicities related to physiological uptake. Therefore, building on our previous promising results obtained with bismuth-213 or during the imaging of MM with copper-64, we have once more favored CD138 as a target antigen [21,22,23,49]. CD138 or syndecan-1 has long been considered as the most reliable marker of MM and is still widely used for the diagnosis of MM. Among all immune cells, this molecule is specifically expressed by mature B-cells only. Furthermore, CD138 has been targeted with Chimeric Antigen Receptor T cells in a clinical trial in an MM context [50]. Although this molecule is expressed on all epithelial cells, the skin data in Figure 2 show that 9E7.4 mAb cannot reach epithelial cells in the early stage of injection. So, considering the short half-life of astatine-211, the major part of the activity is deposited on accessible cells, including plasma cells. However, targeting mouse CD138 with the 9E7.4 antibody results in a rapid liver uptake. This uptake appears to be a specific binding with immunofluorescence labeling (Figure 2c). Furthermore, this hepatic binding did not follow the specific binding kinetics usually found with an antibody. Indeed, the binding is rapid (33% 15 min post-injection) and decreases over time (16%, 21 h post-injection), potentially due to hepatic metabolism. The derived absorbed dose to the liver is 8 Gy for the most effective dose of 740 kBq. Applying a RBE or RBE2(α/β) factor of 4–5 [51], to take into account the high cytotoxicity of alpha particles as compared to photons, leads to an absorbed dose to the liver superior to 26 Gy, considered as a toxicity threshold for human external beam radiotherapy protocols. However, in the present study, ALAT level, measured 150 days after treatment, indicated values within the normal range for a C57BL6 mouse [27]. Concerning ASAT, levels measured 150 days after TAT are equivalent at all injected activities. In addition, we were unable to observe any histological abnormalities within the liver. This result is in contradiction with a previous study we published, in which we had shown histological but non-enzymatic differences (ASAT, ALAT) as early as 1.4 Gy on nude mice that were injected with ^213^Bi-BSA [52]. We have no explanation for this apparent discrepancy. Different microscopic absorbed dose distributions within the liver could play a role here since BSA remains mainly confined into the blood compartment whereas the anti-mCD138 mAb exhibits a specific binding to the liver. Finally, although no radiation-induced tumors were observed on surviving mice during autopsies, stochastic effects will need to be investigated in the event of a transition to the clinic.

Early hematological toxicity was monitored and was shown to be proportional to the activity administered. From 370 kBq to 740 kBq, this toxicity was transient and did not affect the parameters more than 25 days after treatment. For the lethal activity of 1110 kBq, a nearly complete depletion of white blood cells (WBC) 3 days after therapy was observed (835 +/− 289 WBC/mm^3^) but a rise was seen as early as 4 days later. While the activities of 740 and 1110 kBq led to a decrease in red blood cells count, only the activity of 740 kBq resulted in a rise in the red blood cell rate to a level close to that before the therapy. The times and activities of occurrence of radiotoxicity are in agreement with the literature [19].

Compared to the various studies we have conducted with bismuth-213 [21,22], TAT with astatine-211 showed similar results in terms of survival at 150 days. The study by Chérel et al. showed a mouse survival of 70% with bismuth-213 but described a long-term hepatic toxicity with an increase of ASAT and ALAT. It must however be pointed out that this study was conducted with a different anti-mCD138 mAb that had a higher uptake in the liver. Herein, treatment with astatine-211 demonstrated a comparable overall cure rate (65% vs 70%) without histological alterations or major enzymatic problems.

Regarding the benefits of the treatment demonstrated in this preclinical study, the question of the place of this new strategy into the clinical therapeutic arsenal may be asked. Firstly, we must keep in mind that TAT should be considered in clinical settings to eradicate MRD in order to avoid relapse. This new therapy must not change the current therapeutic protocols based on chemotherapies that allow us to decrease the tumor burden and provide a therapeutic window under the scope of alpha particle emitters. Concerning the vector that can drive alpha particle emitters, several anti-human CD138 antibodies are already routinely used for diagnosis (notably, the B-B4 clone that helped us to produce the 9E7.4). Indeed, we used the recognized sequence by B-B4 to design a similar region into the mouse CD138 sequence to immunize the rat and obtain 9E7.4. Furthermore, the B-B4 clone has already been used in humans to perform a feasibility study on radioimmunotherapy using beta emitters [53]. Nevertheless, a dosimetric study will have to be conducted and adapted to the particularity of astatine-211. Results from the clinical trial (NCT03128034) from Fred Hutchinson Cancer Research Center (Seattle, WA, USA) should help us to determine the tolerated activities, too. To date, results issued from this clinical phase I/II study enrolling patients with acute myeloid leukemia and acute lymphoblastic leukemia for treatment with an anti-CD45 antibody, ^211^At-BC8-B10, before donor stem cell transplant, have not yet been published.

## 4. Materials and Methods 

### 4.1. Mice

Female C57BL/KaLwRij mice, 9 to 11 weeks old at the beginning of the experiments, were purchased from Envigo and housed under conventional conditions in the experimental therapeutic unit of IRS UN (Institut de Recherche en Santé de l’Université de Nantes). Experiments performed in this study were approved by the local veterinary services (license no. B44.565). 

### 4.2. 5T33 Cell Line

The 5T33 murine MM cell line was kindly provided by Dr. Harvey Turner (Nuclear Medicine Service, Fremantle Hospital, Western Australia) with the permission of Dr. Jiri Radl (TNO Institute, Leiden, Netherlands). Cells were cultured in RPMI 1640 medium (Gibco) containing 2 mM L-glutamine (Gibco) and 10% heat-inactivated fetal calf serum (PAA Laboratories/GE Healthcare Europe GmbH, Cölbe, Germany) at 37 °C, 5% CO_2_, 95% humidity.

### 4.3. Antibodies

At our request, the anti-CD138 mAb, 9E7.4, was produced by GeneCust (Dudelange, Luxembourg) by immunization of a rat with a 40-amino-acid peptide derived from the murine CD138 protein. The 9E7.4 mAb was a rat IgG2a, κ and presented a Kd about 10^−10^ M. For cytometry experiments and for immunofluorescence experiments, 9E7.4 mAb was respectively labeled with Dylight 650 and with Alexa Fluor 647 with a protein labeling kit provided by Life Technologies according to the supplier’s protocol.

The isotype control used for the therapy was a rat IgG2a, κ purchased from R&D Systems.

The labeling Alexa Fluor 647-isotype control IgG2a, κ for the immunofluorescence experiments was purchased from Biolegend (San Diego, CA, USA).

### 4.4. Flow Cytometry

The binding of the ^211^At radiolabeling 9E7.4 mAb was assessed by flow cytometry. MM 5T33 cells were incubated for 1h with conjugated 9E7.4 or unmodified 9E7.4 and washed 3 times in PBS-BSA 0.1%. Then, secondary antibody anti-Rat-PE (Jackson Immunoresearch) was added and incubated for 1h. Three washings were performed and cells were analyzed on a FACSCalibur (BD Biosciences, Le Pont de Claix, France). Data were analyzed using FlowJo software (Becton Dickinson & Company, Franklin Lakes, NJ, USA).

### 4.5. ^211^At Antibody Radiolabeling

^211^At was produced at the Arronax cyclotron facility and provided as a chloroform solution as reported elsewhere [54]. From this solution, the activity needed was evaporated to dryness and redissolved in 10 mg/mL aqueous sodium sulfite providing the [211At] NaAt species necessary for the radiolabeling.

^211^At-9E7.4 mAb and ^211^At-isotype control were then produced in two steps from a biaryliodonium salt precursor of *N*-succinimidyl-3-[211At]astatobenzoate (SAB) as reported previously [25]. Briefly, to succinimidyloxycarbonyl)phenyl(4-methoxyphenyl)iodonium triflate (2.5 mM in CH_3_CN, 190 µL) we added [211At]NaAt (≈ 100 MBq, 10 µL) and the solution was heated for 30 min at 60 °C. SAB was then isolated by passing the reaction solution through a disposable Sep-Pak Vac 3cc (500 mg) silica cartridge (Waters) using ethyl acetate as eluent. After evaporation of AcOEt to dryness under a gentle stream of nitrogen, SAB was dissolved in 10 µL DMSO and 9E7.4 mAb or isotype control was added (concentrated adjusted at 5 mg/mL^-1^ in borate buffer (0.3M, pH 8.7) using a disposable Amicon Ultra-4 centrifugal unit (Millipore)). The solution was incubated for 30 min at 20 °C. Conjugation yields were assessed by silica gel impregnated paper strips (ITLC-SG) analysis of an aliquot using MeOH as eluent and a Cyclone phosphorimaging scanner. The unbound SAB was eliminated using a PD-10 size exclusion chromatography column (Sephadex G25, GE Healthcare, Little Chalfont, UK) and PBS as eluent. The radiochemical purity was finally assessed by ITLC-SG.

### 4.6. Immunoreactivity Assay

The immunoreactivity of ^211^At-9E7.4 mAb was assessed using magnetic beads (Pierce, Thermo Scientific, Waltham, Mass., USA) labeled with a 40-amino-acid peptide recognized by the 9E7.4 antibody. One picomole of ^211^At-9E7.4 mAb in 100 µL of PBS-BSA 0.1% was incubated 15 min at room temperature with 20 µL of coated magnetic beads (10 mg/mL). Using a magnetic rack, supernatants containing non-reactive antibodies and magnetic beads containing reactive antibodies were collected separately and counted on a gamma counter (Perkin Elmer). The immunoreactivity was 83.0 ± 3.0%.

### 4.7. ^211^At-9E7.4 Biodistribution

Two days before administration of the radioimmunoconjugate, Lugol’s 1% solution was added to the drinking water of the mice (0.1 mL/L). Mice received 370 kBq of ^211^At-9E7.4 mAb at a specific activity of 85 MBq/mg. Fifteen minutes, 1 h, 4 h, 7 h, 14 h and 21 h after radioimmunoconjugate injection, blood sample, neck (for thyroid), liver, kidney, gut, lungs, muscle, spleen, skin, brain, heart, flat bone, femur, and stomach were collected, weighed and counted on a calibrated and normalized gamma counter (Perkin Elmer, Waltham, MA, USA). The experiment was approved by the local veterinary committee (APAFIS #6145) and carried out in accordance with relevant guidelines and regulations.

### 4.8. Immunofluorescence

In order to study the specific binding of the 9E7.4 antibody on the liver, 10 µm frozen liver sections were stained with the 9E7.4 antibody labeled with Alexa Fluor 647. Briefly, frozen sections were fixed in 4% paraformaldehyde for 15 min, washed 3 times with PBS, incubated with a normal rat serum for 10 min and incubated overnight with the 9E7.4 mAb or with the isotype control IgG2a, κ labeled with Alexa Fluor 647 at 5 µg/mL^−1^. The sections were washed 3 times with PBS and incubated with a DAPI solution at 30 µM for 5 min. After 3 washes in PBS, sections were mounted in Prolong gold (Life technologies Carlsbad, CA, USA) and scanned using a slide scanner, Nanozoomer Hamamatsu^®^.

### 4.9. ^211^At-9E7.4 TAT Study

C57BL/KaLwRij mice received one million 5T33 cells into a tail vein 10 days before TAT. Two independent studies were performed.

Two days before administration of the radioimmunoconjugate, Lugol’s 1% solution was added to the drinking water of the mice (0.1 mL/L).

In the first study, groups of 6 mice each were injected via the tail vein with PBS (control group), 370, 740 or 1110 kBq of ^211^At-9E7.4 at a specific activity of 89 MBq/mg.

In the second study, groups of 10 mice each were injected via the tail vein with PBS, 370, 555 or 740 kBq of ^211^At-9E7.4 at a specific activity of 94.9 MBq/mg. An isotype control group (*n* = 10) was injected with 555 kBq of ^211^At-IgG2a, κ at a specific activity of 28.3 MBq/mg. Body weights were measured every week and blood samples were collected by retroorbital bleeding to assess blood counts (Melet-Schloesing Laboratories Osny, France), renal and hepatic function and level of 5T33-secreted immunoglobulin. Mice were sacrificed when the body weight loss was greater than 20% of initial body weight or when the mice presented signs of pain, signs of paraplegia or extramedullary tumor mass. The experiment was approved by the local veterinary committee (APAFIS #7414) and carried out in accordance with relevant guidelines and regulations.

### 4.10. Serum Toxicity Assessments

Serum was assayed for kidney function by measuring creatinine level using the assay kit from SIGMA-ALDRICH. To test hepatic function, serum aspartate aminotransferase (ASAT) and alanine aminotransferase (ALAT) activities were measured using kits from SIGMA-ALDRICH. 

### 4.11. Histological Examination of Mouse Organs

Mice injected with 370 kBq (*n* = 2), 555 kBq (*n* = 2) and 740 kBq (*n* = 3) were sacrificed 160 days after injection, for histological examination. Two age-matched mice were used as control. The liver, kidney and spleen from each mouse were fixed in 4% neutral-buffered formalin and processed by routine methods. Liver and spleen sections were stained with Hematoxylin-Phloxin-Saffron (HPS), Periodic Acid Schiff (PAS) and Sirius Red (SR) by Cellular and Tissular Imaging Core Facility of Nantes University (MicroPICell). Kidney sections were stained with HPS, PAS and Masson’s Trichrome (MT). All slides were scanned using a slide scanner, Nanozoomer Hamamatsu^®^ and evaluated by a certified pathologist. Toxicity was assessed as previously described [52] and as defined by the INHAND Project (International Harmonization of Nomenclature and Diagnostic Criteria for Lesions in Rats and Mice) [28,29].

### 4.12. Quantification of Mouse Myeloma IgG2b

The levels of monoclonal IgG2b produced by 5T33 cells in vivo were measured in the serum of mice by Enzyme-Linked Immunosorbent Assay (ELISA) according to the procedure previously described [21]. Briefly, wells of ELISA microplates were coated overnight with a goat antibody directed against mouse IgG2b (BD Biosciences), washed 3 times with PBS-tween 0.05% and diluted mice serum (1/100,000) was deposited. After 2 h of incubation, 3 washes were performed and a biotin goat antibody directed against another epitope of mouse IgG2b (BD Biosciences) was deposited. Two hours later, wells were washed 3 times and a solution of streptavidin-horseradish peroxidase (R&D) was incubated for 20 min. After 3 washes, the enzyme substrate was added and the reaction was stopped by adding H_2_SO_4_ (2N). Measurements were performed at 405 nm using an ELISA microplate reader.

### 4.13. Dosimetry

Time–activity curves (not corrected for physical decays) were derived from the biodistribution study for each organ and fitted, using PRISM software (Version 7, GraphPad Software, Inc., San Diego, CA, USA), with either a mono-exponential decay function (blood, flat bones, femurs, muscle, liver, kidneys, lungs, spleen, brain, heart) or with the following bi-exponential function (intestine, skin, stomach):

ƒ(t) = A1.[e−λ1.t − e−λ2.t]

with A_1_, λ_1_ and λ_2_ being adjustable parameters.

The time-integrated activity for each organ was then derived by calculating the area under each fitted time–activity curve.

Finally, absorbed doses were calculated assuming a local energy deposition within all organs: time-integrated activities, weighed by the tissue mass, were multiplied by the energy of the emitted α-particles (6.79 MeV per decay, considering the contribution of ^211^At and ^211^Po with the appropriate branching ratio, according to the MIRD radionuclide data and decay schemes) [55].

### 4.14. Statistical Considerations

All data are presented as mean ± standard deviation. Groups were compared using Prism 7.0 (GraphPad Software, Inc., San Diego, CA, USA). Survival curves were compared using a log-rank (Mantel–Cox) test. For toxicity measurements (ASAT, ALAT, creatinine), a non-parametric t-test was performed. *p* values of less than 0.05 were considered statistically significant.

## 5. Conclusions

In the treatment of multiple myeloma, TAT associating the alpha particle emitter, astatine-211, and a full size anti-CD138 antibody appears to be a beneficial combination that provides complete responses in 65% of cases without long-term radiotoxicity. As efficient as bismuth-213, TAT with astatine-211 is a good alternative therapy as its production in cyclotron can prevent the supply problems encountered with actinium-225/bismuth-213 generators.

## Figures and Tables

**Table 1:** Summary of the survival study and cause of death. Numbers in brackets indicate paraplegic mice with extramedullary lesions. Data were issued from 2 independent experiments.

Group	Mice/Group	Surviving Mice	Dead Mice	Cause of Death				
				Acute Toxicity	Paraplegia	Extramedullary Lesions	No Macroscopic Lesion but High Serum IgG Level	Undetermined Death
Control	16	0	16	-	7	9 (2)	1	1
9E7.4 at 370 kBq	16	2	14	-	4	10 (2)	-	2
9E7.4 at 555 kBq	10	3	7	-	4	5 (3)	-	1
9E7.4 at 740 kBq	16	10	6	-	3	4 (1)	-	-
9E7.4 at 1110 kBq	6	0	6	6	-	-	-	-
Isotype control at 555 kBq	10	0	10	-	5	5 (1)	1	-

**Table 2:** ^211^At-9E7.4 dosimetry study. Mean absorbed doses, expressed in Gy/MBq, and their standard error for the different organs.

Organs	Absorbed Dose (Gy/MBq)	SE (Gy/MBq)
Blood	8.0	0.7
Flat Bone	1.5	0.2
Liver	10.9	1.0
Kidneys	3.6	0.3
Gut	6.6	1.3
Lungs	5.8	0.8
Muscle	0.8	0.3
Spleen	8.2	1.7
Skin	2.6	0.9
Brain	0.2	0.0
Heart	3.8	0.6
Femur	1.7	0.3
Stomach	9.5	4.7
